# Giant Cell Myocarditis: A Time Sensitive Distant Diagnosis

**DOI:** 10.7759/cureus.6712

**Published:** 2020-01-20

**Authors:** Medhat Ghaly, Danise Schiliro, Jadwiga Stepczynski

**Affiliations:** 1 Internal Medicine, Yale School of Medicine, New Haven, USA; 2 Internal Medicine, Yale-Waterbury Internal Medicine Residency Program, Yale School of Medicine, Waterbury, USA

**Keywords:** giant cell myocarditis, cardiac arrhythmia, cardiomyopathy, congestive heart failure, sudden cardiac death, aicd, cardiac mri, ventricular tachycardia, endomyocardial biopsy

## Abstract

Giant cell myocarditis is a rare type of rapidly progressive myocarditis. We present a dramatic case of giant cell myocarditis in a young female with an initial presentation of acute heart failure. Her clinical course was complicated with recurrent cardiac arrhythmias, specifically non-sustained ventricular tachycardia, for which a dual chamber automated implantable cardioverter defibrillator (AICD) was implanted. Eventually, she presented with cardiac arrest despite being on antiarrhythmic medication and an implantable defibrillator. In the right clinical context, such as an acute presentation of unexplained new-onset heart failure and arrhythmias in a young patient, it is very important to maintain high suspicion of such a rare disease.

## Introduction

This case highlights a rapidly progressive type of myocarditis. The incidence of giant cell myocarditis (GCM) has been reported to range from 0.007% to 0.051% in a large autopsy study, however, this number might be underestimated as autopsy is not routinely performed for all unexplained sudden cardiac death. Without appropriate immunosuppressive therapy, the median survival from GCM symptom onset to death or transplant is only three months. The most common initial manifestations are congestive heart failure symptoms, ventricular arrhythmias, and atrioventricular block, but rarely may also present as sudden cardiac death. The definitive diagnosis depends on the pathologic findings obtained by endomyocardial biopsy, however, due to the invasiveness of the procedure, it is often not done early enough, and results are thus delayed [1].

## Case presentation

**Figure 1:**
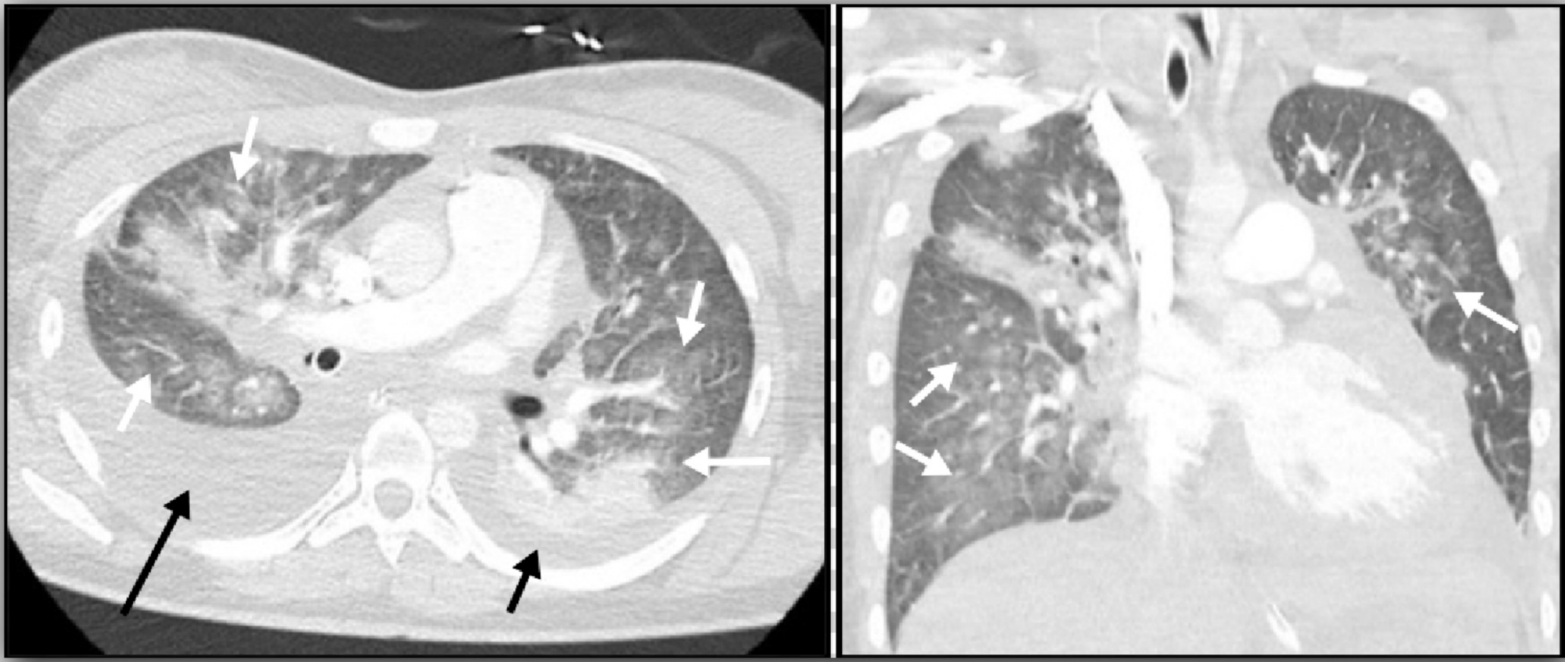
Chest CT scan with intravenous contrast demonstrates evidence of pulmonary edema (white arrows) with small left (short black arrow) and moderate right-sided pleural effusion (long black arrow).

A 26-year-old female with no significant past medical history presented with five days of worsening shortness of breath, orthopnea and chest tightness. Acute hypoxic respiratory failure rapidly developed requiring intubation and mechanical ventilation. The electrocardiogram (ECG) showed sinus rhythm at 80 beats per minute with a normal QRS morphology and no ST segment or T wave changes. On initial laboratory testing, troponin and N-terminal pro-brain natriuretic peptide (NT-pro-BNP) levels were elevated at 10,300 pg/ml. Computed tomography (CT) scan of the chest showed evidence of pulmonary edema and right-sided pleural effusion (Figure 1). Echocardiography revealed a left ventricular ejection fraction (LVEF) of 25% without evidence of valvular dysfunction or apical ballooning.

She was treated aggressively with intravenous (IV) diuretics, and inotropic agents. The working diagnosis initially was viral myocarditis. Cardiac magnetic resonance imaging (MRI) demonstrated severely enlarged left ventricular size, depressed LV systolic function with LVEF of 39%, mild segmental hypokinesis, and multiple mid myocardial patchy areas of increased T2 signal compatible with myocardial edema. There were also many corresponding areas of delayed contrast enhancement involving areas of subepicardial, mid-myocardial, and transmural enhancement with relative sparing of the subendocardium in multiple segments including the anteroseptal, anterior, anterolateral, and inferior walls extending from the base to the apex. These findings increased the likelihood of acute myocarditis as the underlying etiology.

**Figure 2:**
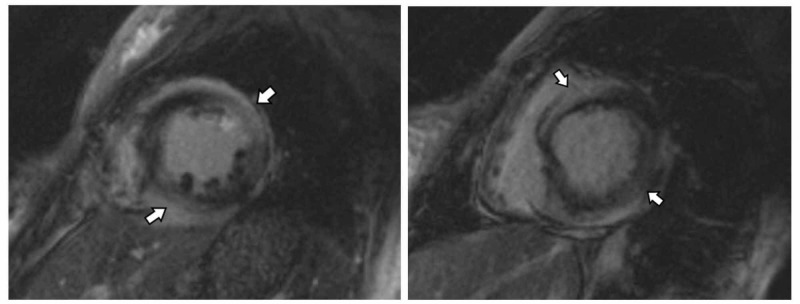
Cardiac MRI in the short axis views demonstrate abnormal delayed gadolinium enhancement (arrows) involving mid-myocardial and sub-epicardial regions of multiple segments of the left ventricle. MRI: Magnetic resonance imaging.

Viral direct florescent antibody tests were negative. She had multiple outpatient visits for medication optimization without notable sustained clinical improvement. One month later, a repeat MRI showed an LVEF of 35%, worsened hypokinesis, and progression of the diffuse patchy myocardial delayed enhancement seen previously (Figure 2).

**Figure 3:**
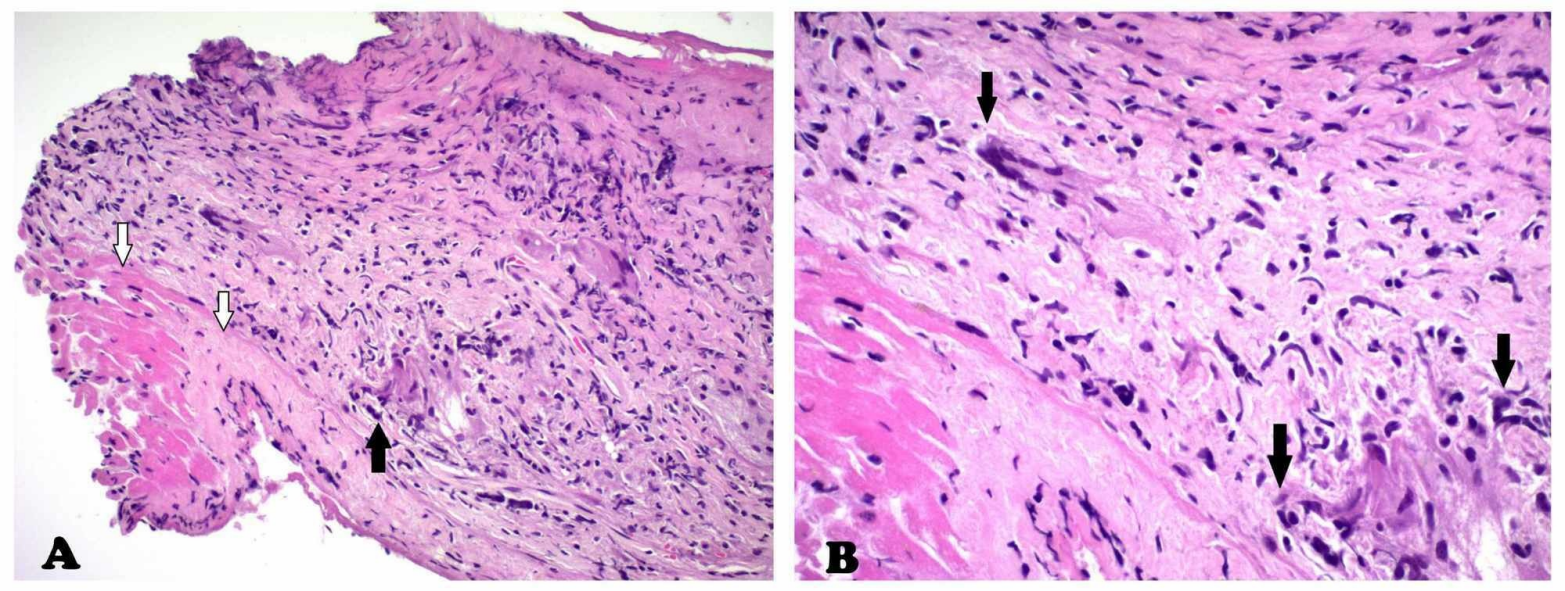
Histopathology findings of endomyocardial biopsy specimen demonstrates exuberant inﬂammatory inﬁltrate consisting predominantly of macrophages and mononuclear cells with collections of multinucleated giant cells (black arrows). Viable myocytes and necrotic tissue are both present (white arrows). The destructive nature of this inﬁltrate is consistent with active giant cell myocarditis. HE stain: (A) 20x, (B) 40x

Deterioration of cardiac function despite standard heart failure therapy in the context of these MRI findings raised concerns for an infiltrative cardiomyopathy. Positron emission tomography (PET) scan was not suggestive of cardiac sarcoidosis and ischemic workup ruled out myocardial ischemia. Right heart catheterization showed normal filling pressures. Endomyocardial biopsy (EMB) was performed. Congo red stain was negative, excluding amyloidosis. Histopathology, however, showed multiple inﬂammatory cells including macrophages and multi-nucleated giant cells with areas of myocardial necrosis consistent with giant cell myocarditis (Figure 3).

Immunosuppressive therapy was initiated, including prednisone, azathioprine and cyclosporine, and there was relative improvement in her heart failure symptoms. Four months after her initial presentation, she developed non-sustained ventricular tachycardia, for which a dual chamber automated implantable cardioverter defibrillator (AICD) was placed and antiarrhythmic therapy was initiated using sotalol. Sotalol was increased to the maximum dose in attempt to control the intermittent non-sustained ventricular arrhythmias with a plan to consider catheter ablation therapy. A few weeks later, and before the ablation procedure could take place, she presented to the ED with cardiac arrest, and was pronounced dead after unsuccessful attempts at resuscitation.

## Discussion

GCM is a rare and rapidly progressive myocarditis that affects young to middle aged adults and is often fatal. It is characterized by a mixed myocardial infiltrate with multinucleated giant cells and cardiac necrosis. Evidence suggests that it arises secondary to immune dysregulation mediated by T lymphocytes [1]. Patients typically present with rapidly progressive congestive heart failure frequently associated with refractory ventricular arrhythmias. GCM should be considered when acute heart failure does not respond to standard treatment or in the presence of refractory electrical storms despite the use of aggressive antiarrhythmic drug therapy. The gold standard for diagnosis is endomyocardial biopsy. Cardiac MRI and PET are useful for identifying targets for biopsy [2]. The combination of MRI and EMB has been shown to improve detection rates. When clinical suspicion remains high, one non-diagnostic biopsy is insufficient to exclude the diagnosis and repeat biopsy is warranted.

In certain clinical situations, there is a role for early EMB where results may meaningfully estimate prognosis or guide treatment. Per the American College of Cardiology/American Heart Association (ACC/AHA) guidelines, EMB should be performed in the setting of new onset heart failure with hemodynamic compromise or new ventricular arrhythmias (Class I recommendation) [3].

Survival rates for patients with GCM have improved since the implementation of immunosuppressive regimens and the use of mechanical circulatory devices as bridges to cardiac transplantation. Patients treated with combined immunosuppression have an average survival of 12.3 months from the onset of symptoms, compared to 3.0 months without immunosuppression [4,5]. GCM confers high cardiovascular mortality and is associated with a high cardiac transplant rate, frequently necessitating bridging mechanical circulatory support. It has been suggested that aggressive and early arrhythmia management, including radiofrequency catheter ablation, may play a role in prolonging survival. Early initiation of immunosuppressive therapy is crucial to improved prognosis and increased transplant-free survival [6]. While heart transplant is the only available definitive treatment, recurrence of GCM after transplant has been reported [4]. In this case, immunosuppression appeared to improve her heart failure symptoms, and for that reason, transplant evaluation was initially delayed.

**Figure 4:**
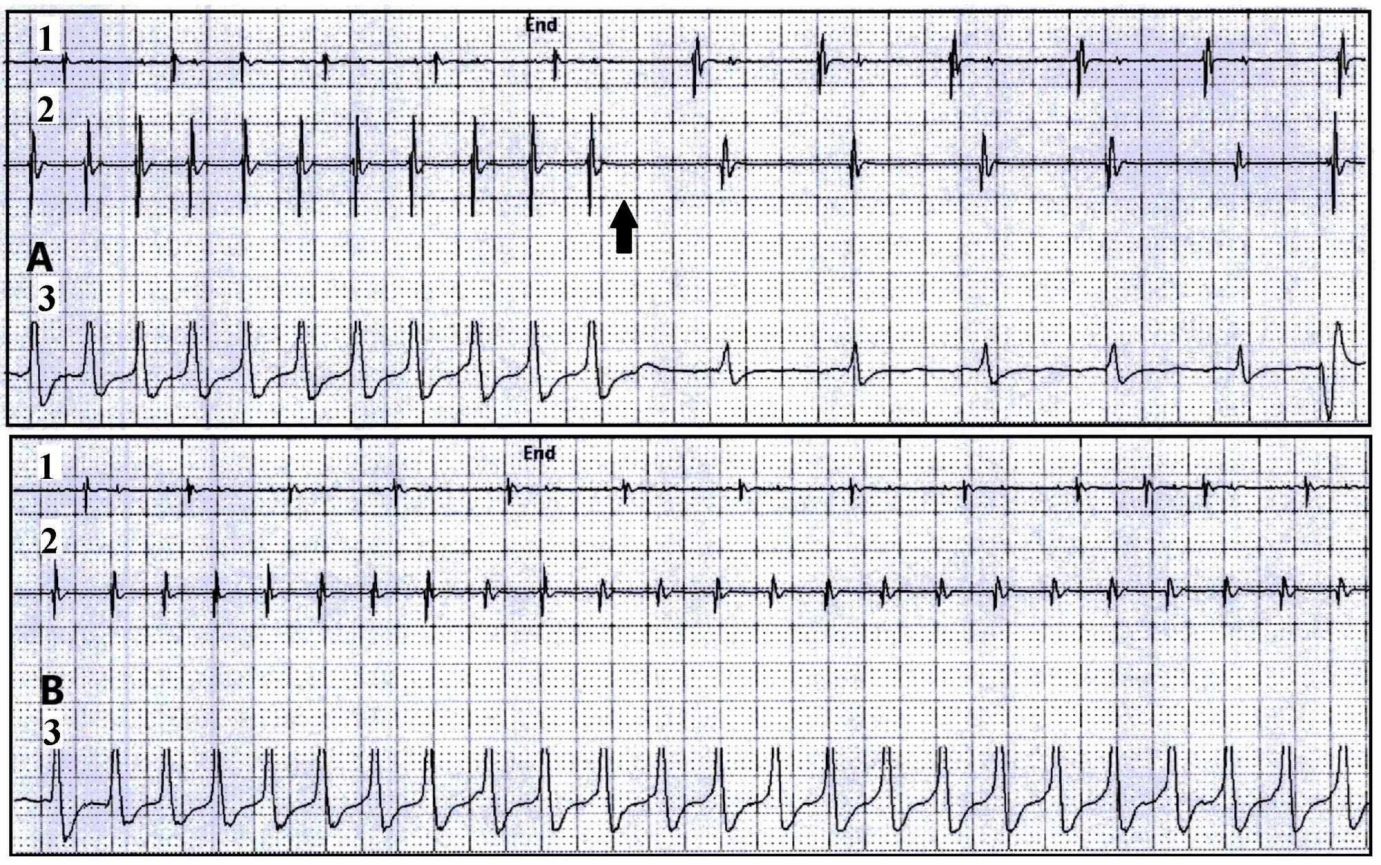
AICD device interrogation: Two rhythm strips from AICD interrogation demonstrate examples of two different episodes of non-sustained ventricular tachycardia (NSVT). (Panel A: Episode of NSVT with HR 186 bpm, spontaneously terminated [black arrow], this episode lasted for 14 seconds. Panel B: NSVT with HR 194 bpm, episode lasted for 19 seconds). (1) AICD atrial lead tracing. (2) AICD ventricular lead tracing. (3) Surface ECG tracing. AICD: Automated implantable cardioverter defibrillator; HR: Heart rate; bpm: beat per minute.

Recurrent non-sustained ventricular arrhythmias persisted, necessitating the use of higher doses of anti-arrhythmic medications (Figure 4).

Myocarditis generally is associated with different types of potentially life-threatening arrhythmias that may present at any stage of the disease clinical course. Arrhythmias may represent underlying electrical instability of the myocardium. Ventricular arrhythmias are more commonly associated with giant cell myocarditis with a prevalence of 55% [7]. Arrhythmias may result from active inflammation, myocardial infiltration by giant cells, or residual myocardial scarring as sequelae of chronic inflammation. The latter has been observed in patients with sarcoidosis and myocardial infarction wherein the arrhythmias are related to myocardial scarring over disease activity [2]. The risk of sudden cardiac death in myocarditis does not correlate with the severity of myocardial inflammation and myocardial healing is not necessarily associated with the disappearance of arrhythmias [8]. It is thought that refractory ventricular arrhythmia ultimately led to the demise of this patient.

## Conclusions

GCM is a rare and rapidly progressive myocarditis that affects young to middle aged adults and is often fatal. Maintaining a high index of suspicion in the appropriate clinical settings is critical in enabling early diagnosis. Early diagnosis allows for early initiation of targeted therapy such as immunosuppressive therapy which translates into improved survival. In this case, the patient’s heart failure symptoms were minimal on medical management. Cardiac arrhythmia was the suspected cause of death. This highlights the importance of early diagnosis and the need for aggressive arrhythmia management, including early consideration of radiofrequency catheter ablation therapy because of the known risk of potential life threatening arrhythmias in this patient population.
